# Effects of remote ischemic preconditioning (RIPC) and chronic remote ischemic preconditioning (cRIPC) on levels of plasma cytokines, cell surface characteristics of monocytes and in-vitro angiogenesis: a pilot study

**DOI:** 10.1007/s00395-021-00901-8

**Published:** 2021-10-14

**Authors:** Lars Hummitzsch, Karina Zitta, Lena Fritze, Jonas Monnens, Patrick Vollertsen, Matthias Lindner, Rene Rusch, Katharina Hess, Matthias Gruenewald, Markus Steinfath, Fred Fändrich, Rouven Berndt, Martin Albrecht

**Affiliations:** 1 Department of Anesthesiology and Intensive Care Medicine, University Hospital Schleswig-Holstein, Kiel, Germany; 2 Department of Cardiovascular Surgery, University Hospital Schleswig-Holstein, Kiel, Germany; 3 Department of Pathology, University Hospital Schleswig-Holstein, Kiel, Germany; 4 Department for Applied Cell Therapy, University Hospital Schleswig-Holstein, Kiel, Germany

**Keywords:** Remote ischemic preconditioning (RIPC), Chronic remote ischemic preconditioning (cRIPC), Cardiac protection, Monocytes, Tie-2, Angiogenesis, Cardiovascular disease

## Abstract

Remote ischemic preconditioning (RIPC) protects the heart against myocardial ischemia/reperfusion (I/R) injury and recent work also suggested chronic remote ischemic conditioning (cRIPC) for cardiovascular protection. Based on current knowledge that systemic immunomodulatory effects of RIPC and the anti-inflammatory capacity of monocytes might be involved in cardiovascular protection, the aim of our study was to evaluate whether RIPC/cRIPC blood plasma is able to induce in-vitro angiogenesis, identify responsible factors and evaluate the effects of RIPC/cRIPC on cell surface characteristics of circulating monocytes. Eleven healthy volunteers were subjected to RIPC/cRIPC using a blood pressure cuff inflated to > 200 mmHg for 3 × 5 min on the upper arm. Plasma and peripheral blood monocytes were isolated before RIPC (Control), after 1 × RIPC (RIPC) and at the end of 1 week of daily RIPC (cRIPC) treatment. Plasma concentrations of potentially pro-angiogenic humoral factors (CXCL5, Growth hormone, IGFBP3, IL-1α, IL-6, Angiopoietin 2, VEGF, PECAM-1, sTie-2, IL-8, MCSF) were measured using custom made multiplex ELISA systems. Tube formation assays for evaluation of in-vitro angiogenesis were performed with donor plasma, monocyte conditioned culture media as well as IL-1α, CXCL5 and Growth hormone. The presence of CD14, CD16, Tie-2 and CCR2 was analyzed on monocytes by flow cytometry. Employing in-vitro tube formation assays, several parameters of angiogenesis were significantly increased by cRIPC plasma (number of nodes, *P* < 0.05; number of master junctions, *P* < 0.05; number of segments, *P* < 0.05) but were not influenced by culture medium from RIPC/cRIPC treated monocytes. While RIPC/cRIPC treatment did not lead to significant changes of the median plasma concentrations of any of the selected potentially pro-angiogenic humoral factors, in-depth analysis of the individual subjects revealed differences in plasma levels of IL-1α, CXCL5 and Growth hormone after RIPC/cRIPC treatment in some of the volunteers. Nevertheless, the positive effects of RIPC/cRIPC plasma on in-vitro angiogenesis could not be mimicked by the addition of the respective humoral factors alone or in combination. While monocyte conditioned culture media did not affect in-vitro tube formation, flow cytometry analyses of circulating monocytes revealed a significant increase in the number of Tie-2 positive and a decrease of CCR2 positive monocytes after RIPC/cRIPC (Tie-2: cRIPC, *P* < 0.05; CCR2: RIPC *P* < 0.01). Cardiovascular protection may be mediated by RIPC and cRIPC via a regulation of plasma cytokines as well as changes in cell surface characteristics of monocytes (e.g. Tie-2). Our results suggest that a combination of humoral and cellular factors could be responsible for the RIPC/cRIPC mediated effects and that interindividual variations seem to play a considerable part in the RIPC/cRIPC associated mechanisms.

**Supplementary Information:**

The online version contains supplementary material available at 10.1007/s00395-021-00901-8.

## Introduction

Remote ischemic preconditioning (RIPC) for organ protection is established by applying brief episodes of ischemia and reperfusion in distant tissues or organs (e.g. upper or lower limb). Over the last decades many experimental and also some smaller clinical studies have proven cardioprotective effects of RIPC, especially in the context of myocardial ischemia/reperfusion (I/R) injury [2, 18, 40]. In clinical studies using patients with ST-elevation myocardial infarction (STEMI), remote ischemic conditioning (RIC) increased myocardial salvage and reduced myocardial infarct size by 20–30% or resulted in fewer cardiac deaths or hospitalizations for heart failure when applied before or during reperfusion [10, 12, 43, 50]. Interestingly, large outcome trials could mostly not confirm the promising results from the initial proof of principle studies [14, 15, 18, 34]. One explanation for this observation, which applies to at least 2 large studies that investigated the effects of RIPC on clinical outcomes in patients undergoing coronary-artery bypass graft (CABG), is that patients were anesthetized with propofol [14, 34], which has been described to abrogate the protection by RIPC [28].

In addition to the external factors such as the type of anesthesia, patient related confounders, such as age, co‐morbidities (e.g. diabetes, hypertension, hyperlipidemia etc.) and co‐medications (e.g. β‐blockers, calcium antagonists, statins, nitrates etc.) are also discussed to be responsible for the inconsistent results regarding beneficial effects of RIPC [20, 25, 26]. In this context it has been assumed, that diseases such as diabetes and various medication can increase the conditioning threshold, requiring a more robust conditioning signal to induce protective effects of RIPC [33]. Hence, recent studies suggested that daily repeated RIPC (chronic RIPC; cRIPC) could possibly overcome this problem and might be more effective for cardiovascular protection than a single RIPC application [6].

The inflammatory immune response plays an important role in the development of I/R injury and determines the dimension of tissue injury after myocardial infarction. In the early phase of I/R injury, pro-inflammatory cells like neutrophils and monocytes are recruited into the site of infarction, clearing necrotic cell debris, and secreting pro-inflammatory cytokines [1]. This pro-inflammatory response recruits further leukocytes and amplifies the inflammation within the infarction area beyond the viable border zone of infarction [37]. Therefore, besides humoral factors and nerval pathways, systemic immunomodulatory effects of RIPC might be crucial for RIPC mediated cardio protection as injury progression and repair processes are profoundly influenced by peripheral immunity [2, 16, 32, 49]. In contrast, enhanced inflammation is associated with exacerbated ischemic outcome, but post-ischemic inflammation is also considered to be a necessary process for tissue remodeling. Employing an animal model of post-stroke remote ischemic limb conditioning Yang et al. demonstrated a shift of circulating monocytes to a CCR2 positive pro-inflammatory monocyte subset resulting in reduced acute brain injury, swelling, and improved motor/gait function suggesting that pro-inflammatory monocytes are able to attenuate acute injury and promote functional recovery in chronic stroke [49]. In the heart it has been shown that monocytes undergo dynamic changes in their polarization after myocardial infarction. At first, pro-inflammatory monocytes migrate into the infarction area secreting pro-inflammatory cytokines (e.g. TNF-α, IL-1β). Consequently, increased levels of circulating pro-inflammatory monocytes after myocardial infarction are associated with larger myocardial injury and reduced left ventricular function [45, 46]. However, monocytes switch their phenotype into an anti-inflammatory and proliferative cell type 3–4 days after myocardial infarction promoting healing processes by inducing neo-angiogenesis and collagen production [1, 37, 39].

Based on current knowledge, humoral factors as well as monocytes could play an important role in RIPC/cRIPC mediated cardiovascular protection. The aim of our study was therefore to evaluate whether RIPC/cRIPC blood plasma from RIPC/cRIPC treated healthy volunteers is able to induce in-vitro angiogenesis, identify responsible factors and evaluate the effects of RIPC/cRIPC on cell surface characteristics of circulating monocytes.

## Materials and methods

### Study design and experimental setting

**Fig. 1:**
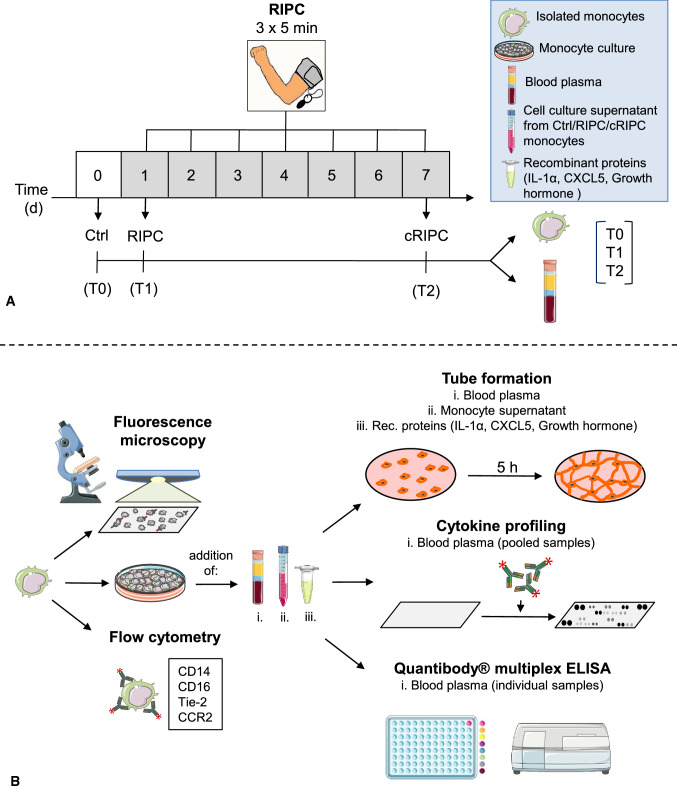
Experimental design of the study. **A** in-vivo part. **B** in-vitro part. *Ctrl* control

The study was approved by the local ethics committee of the Christian-Albrechts University Kiel, Germany (D552/18) and was performed in accordance with the Declaration of Helsinki and the Medical Research Involving Human Subjects Act. Eleven healthy volunteers (Table 1) were subjected to a RIPC/cRIPC procedure using a blood pressure cuff inflated to > 200 mmHg for 3 × 5 min on the upper arm. As several studies suggest that high-intensity exercise preconditioning elicits cardioprotection similar to RIPC [35], the fitness level of the subjects was determined on the basis of a 3-level scale (high-moderate-low) and the assignment to a respective level was made based on an anamnesis interview by the responsible physician. Plasma and peripheral blood monocytes were isolated before RIPC (Control), 3 h after 1 × RIPC (RIPC) and at the end of 1 week of daily RIPC (cRIPC) treatment. Donor plasma and monocyte conditioned culture media were subjected to proteome profiling for cytokine secretion and/or Quantibody® array-based multiplex ELISA analyses as well as tube formation assays for in-vitro angiogenesis. The presence of CD14, CD16, Tie-2 and CCR2 was analyzed on monocytes by flow cytometry (Fig. 1).

**Table 1:** Demographic data of volunteers involved in the study

Volunteer (internal #)	Age (years)	Gender	Smoking	Infections during study period	Level of fitness
P3	21	Female	No	No	Low
P4	21	Male	No	No	High
P5	21	Female	No	No	Moderate
P6	29	Female	No	No	Moderate—high
P8	26	Male	No	No	Moderate
P9	34	Male	No	No	Moderate
P10	25	Male	No	No	High
P11	23	Female	No	No	High
P12	48	Male	No	No	Moderate
P13	24	Male	No	No	Moderate
P15	28	Male	No	No	Moderate

### Isolation of circulating blood monocytes

Citrate blood was centrifuged for 30 min at 400×*g*. After the isolation of plasma, the buffy coat was resuspended in RPMI-1640 culture medium. The cell suspension was pipetted on Ficoll and was centrifuged for 40 min at 760×*g*. The resulting buffy coat was mixed with RPMI-1640 and centrifuged for 10 min at 760×*g*. This washing step was repeated 3 times. The resulting cells were sorted using the Miltenyi pan monocytes isolation kit (Miltenyi Biotec, Bergisch-Gladbach, Germany), containing a cocktail of biotin-conjugated monoclonal antibodies against antigens not expressed on human monocytes (negative sorting) as described in the manufacturer´s protocol. Briefly, 10 million cells were incubated at 4 °C for 5 min with FcR blocking reagent (human IgG) and the cocktail of biotin conjugated antibodies. Magnetic microbeads conjugated to monoclonal anti-biotin antibodies were added to the solution, incubated for 10 min at 4 °C and subjected to a subsequent magnetic cell separation through a magnetic field. Finally, the monocyte fraction was collected and used for cell culture and further analyses.

### In-vitro culture of blood monocytes

Isolated monocytes were seeded into culture plates at 130.000 cells/cm^2^ containing RPMI-1640 culture medium supplemented with 10% male human AB serum (Access Biologicals, S. Dartmouth, MA, USA). After 1 h incubation at 37 °C, medium was replaced by fresh RPMI-1640 supplemented with 10% human serum and cells were grown at 37 °C for another 23 h. At the end of the incubation time, cell protein was isolated using RIPA lysis buffer (Qiagen, Hilden, Germany) according to the manufacturer´s protocol. Cell culture supernatants were collected and stored at − 20 °C.

### Secretion of cytokines

As pilot screening method plasma and cell culture supernatants were analyzed for 105 cytokines using the human proteome profiler XL cytokine array kit (R&D Systems, Minneapolis, MN, USA) as described in the manufacturer´s protocol. Briefly, samples from each experimental group were pooled using equal volumes and applied to the array membranes carrying antibodies against the respective cytokines. After incubation, a cocktail of biotinylated antibodies and HRP-streptavidin was added and the signals were visualized by chemiluminescence detection, referring to the manual provided. Photographs of the membranes were taken using the Fusion FX Vilber device (Vilber Lourmat, Eberhardzell, Germany) and signal intensities were analyzed using the ImageJ 1.41 software (NIH). Signals were only considered as relevant and were further analyzed if their intensity was > 10% of the mean intensity of the respective reference spots on the array membrane. Based on the proteome profiler results and recent studies of other groups [13, 38], in a second step plasma from each volunteer was individually analyzed for the concentrations of 11 cytokines [C-X-C motif chemokine 5 (CXCL5), Growth hormone (GH), Interleukin-1alpha (IL-1α), Interleukin-6 (IL-6), Insulin like growth factor binding protein 3 (IGFBP3), Angiopoietin 2 (Ang2), Vascular endothelial growth factor (VEGF), Platelet endothelial cell adhesion molecule-1 (PECAM-1), soluble angiopoietin receptor Tie-2 (sTie-2), Interleukin-8 (IL-8) and Macrophage colony stimulating factor (MCSF)] using a custom made Quantibody® array-based multiplex ELISA system in combination with the Quantitative Proteomics Services provided by RayBiotech (RayBiotech, Peachtree Corners, GA, USA).

### Human umbilical vein endothelial cell culture and tube formation assays

Human umbilical vein endothelial cells (HUVEC) were isolated from umbilical cords as described previously [3, 22] [approval by the local ethics committee of the Christian-Albrechts University Kiel, Germany (D519/18 and D518/13)] and cultured in endothelial cell growth medium ECGM (PromoCell, Heidelberg, Germany) supplemented with 4 μL/mL of endothelial cell growth supplement, 0.1 ng/mL epidermal growth factor, 1 ng/mL basic fibroblast growth factor, 90 μg/mL heparin, 1 μg/mL hydrocortisone (all from PromoCell) and 10% fetal bovine serum (Thermo Fisher, Dreieich, Germany). The cells were maintained in a humidified atmosphere (5% carbon dioxide / 95% air) at 37 °C. Angiogenesis was evaluated using IBIDI cell culture dishes (Ibidi GmbH, Munich, Germany) and the protocol provided by the manufacturer. Briefly, 10.000 HUVEC were seeded on Matrigel™ precoated wells containing the respective culture medium supplemented with 10% of donor plasma, 50% of monocyte cell culture supernatant or different concentrations of the following human recombinant proteins alone or in combination: IL-1α (#200-01A; Peprotech, Cranbury, NJ, USA), CXCL5 (#300–22; Peprotech) and Growth hormone (#100–40; Peprotech). In stimulation experiments with recombinant proteins, corresponding controls consisted of HUVEC cultures which were incubated with pooled control plasma (10%) from the 11 donors. The corresponding concentrations of IL-1α, CXCL5 and Growth hormone in the control cultures (baseline) were calculated based on the results of the multiplex ELISA experiments and for stimulations, the appropriate amount of recombinant protein was added to the calculated baseline to achieve the desired final concentration. Photomicrographs of the cells were taken after 5 h of culture. Tube formation parameters (e.g. number of meshes, nodes, segments, junctions, etc.) were analyzed using the angiogenesis analyzer tool of the Image J software 1.41 (NIH) [4, 29].

### Immunofluorescence staining

For immunofluorescence staining, monocytes were seeded into culture wells containing glass coverslips. After 1 h attached monocytes were washed with warm PBS and fixed with 3.7% paraformaldehyde for 15 min at room temperature. After a washing step with PBS, signal enhancer ImageIT solution (Invitrogen, Carlsbad, CA, USA) was added for 30 min at room temperature, followed by cell permeabilization with Tween 20 for 15 min at room temperature. Dilutions of primary antibodies were prepared in 1% BSA (anti-Tie-2, 1:100, Abcam, ab24859, Cambridge, UK; anti-CCR2, 1:50, Novus biological, NBP1-48337, Littleton, CO, USA). Samples were incubated with the respective antibody dilution for 1 h at room temperature. After 3 washing steps in PBS, incubation with respective secondary antibodies coupled with Alexa Fluor 488 (1:1.000 in 1% BSA; Thermo Fisher, A32723, Waltham, MA, USA) or rhodamine (1:125 in 1% BSA; Thermo Fisher, #31685) were performed in the dark for 90 min at room temperature. The glass cover slips containing stained monocytes were placed upside down on a drop of mounting solution (DAPI-containing Fluoromount; Invitrogen) located on microscope glass slides. Analyses were performed using a Leica DM2000 microscope (Leica, Wetzlar, Germany) with fluorescence filters for DAPI, FITC and rhodamine.

### Flow cytometry

Flow cytometry was performed using the BD FACSCalibur™ cytometer (BD Biosciences, Franklin Lakes, USA). Specific antibodies and their corresponding isotypes (dilution for all antibodies:1:20) were directly conjugated with fluorescein isothiocyanate (FITC): CD16 (BD Bioscience, #555406) and its isotype (anti-mouse IgG1*k*; BD Bioscience, #555748). Conjugated with allophycocyanin (APC): Tie-2 (R&D, FAB3131A) and its isotype (anti-mouse IgG1; R&D, IC002A) and CD14 (BD Bioscience, #555399) and its isotype (anti-mouse IgG2a; BD Biosciences, #555576). Conjugated with Alexa fluor 647 (AF647): CCR2 (BD Bioscience, #558406) and its isotype (anti-mouse IgG2b; BD Bioscience, #557903). The gating strategy consisted of (i) identification of monocytes based on their size and granularity (FSC/SSC profiles), (ii) exclusion of non-viable cells (7-AAD, BD Biosciences, #559925), (iii) identification of monocytes positive for CD14, CD16, Tie-2 and CCR2 (Supplemental Fig. 1).

### Statistics

The statistics software GraphPad Prism 5.01 for windows (GraphPad Software: San Diego, USA) was used for data analyses. Values are expressed as the median or mean with standard deviation (SD). All data were tested for normality using the Kolmogorov–Smirnov test. In cases normality was not obtained, the data were transformed (arcsine of square root of *x*) and analyzed using one-way ANOVA with Tukey-test, two-tailed *T* test or one-sample *T* test. The existence of a linear correlation between two parameters was estimated using the Pearson correlation coefficient. A *P* value < 0.05 was considered significant.

## Results

### Effects of RIPC/cRIPC plasma and supernatants derived from RIPC/cRIPC monocytes on in-vitro angiogenesis

**Fig. 2:**
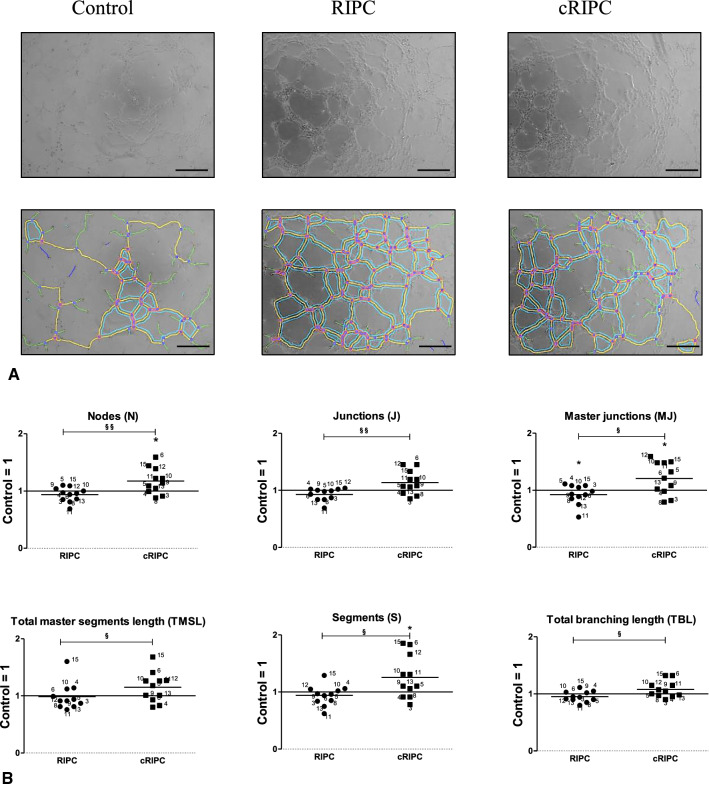
Effects of RIPC/cRIPC plasma on in-vitro angiogenesis. **A** representative images of HUVEC cultures that were incubated with Control, RIPC and cRIPC plasma. Upper panel, original images. Lower panel, graphical results of the analysis using the angiogenesis analyzer tool of the Image J software 1.41 (NIH) [4]. Scale bars depict 300 µm. **B** effects of RIPC and cRIPC plasma on key parameters of tube formation in-vitro. Control values were set to 1. Horizontal lines show the mean. *P < 0.05; one-sample *T* test. ^§^*P* < 0.05; ^§§^*P* < 0.01; two-tailed *T* test

Tube formation assays for angiogenesis were performed with human umbilical vein endothelial cells (HUVEC) that were grown on Matrigel coated dishes. The results show that several parameters of angiogenesis were significantly increased by RIPC/cRIPC plasma (Fig. 2) but were not influenced by media from RIPC/cRIPC treated monocytes (data not shown).

In detail RIPC/cRIPC plasma resulted in changes (relative to Control) of the following parameters, which are regarded as markers for in-vitro angiogenesis: Number of nodes (N): RIPC, 0.94 ± 0.13; cRIPC, 1.18 ± 0.23; cRIPC vs. Control, *P* < 0.05; cRIPC vs. RIPC, *P* < 0.01. Number of junctions (J): RIPC, 0.93 ± 0.11; cRIPC, 1.14 ± 0.21; cRIPC vs. RIPC, *P* < 0.01. Number of master junctions (MJ): RIPC, 0.92 ± 0.17; cRIPC, 1.21 ± 0.29; RIPC and cRIPC vs. Control, *P* < 0.05; cRIPC vs. RIPC, *P* < 0.05. Total master segments length (TMSL): RIPC, 0.99 ± 0.24; cRIPC, 1.15 ± 0.26; cRIPC vs. RIPC, *P* < 0.05. Number of segments (S): RIPC, 0.94 ± 0.18; cRIPC, 1.26 ± 0.37; cRIPC vs. Control, *P* < 0.05; cRIPC vs. RIPC, *P* < 0.05. Total branching length (TBL): RIPC, 0.95 ± 0.09; cRIPC, 1.08 ± 0.14; cRIPC vs. RIPC, *P* < 0.05 (Fig. 2).

### Effects of RIPC/cRIPC on levels of plasma cytokines

In a first step and as pilot approach, we investigated whether RIPC and cRIPC affect the relative levels of blood plasma cytokines. Therefore, pooled donor plasma was screened for 105 cytokines using proteome profiling arrays. 57/105 (54.3%) of the cytokines revealed signal intensities below the defined threshold and were therefore not included into further analyses. RIPC decreased the amount of 9/48 (18.8%) of analyzed cytokines in plasma. 9/48 (18.8%) of the investigated factors were increased, while 30/48 (62.5%) remained unaffected. The effect of cRIPC was more pronounced: 54/105 (51.4%) cytokines revealed signal intensities below the defined threshold and were not included into further analyses. cRIPC decreased the amount of 1/51 (2.0%) of analyzed cytokines in plasma. 49/51 (96.0%) of the investigated factors were increased, while 1/51 (2.0%) remained unaffected. For details on the RIPC/cRIPC regulated plasma proteins please refer to Supplemental Fig. 2.

**Fig. 3:**
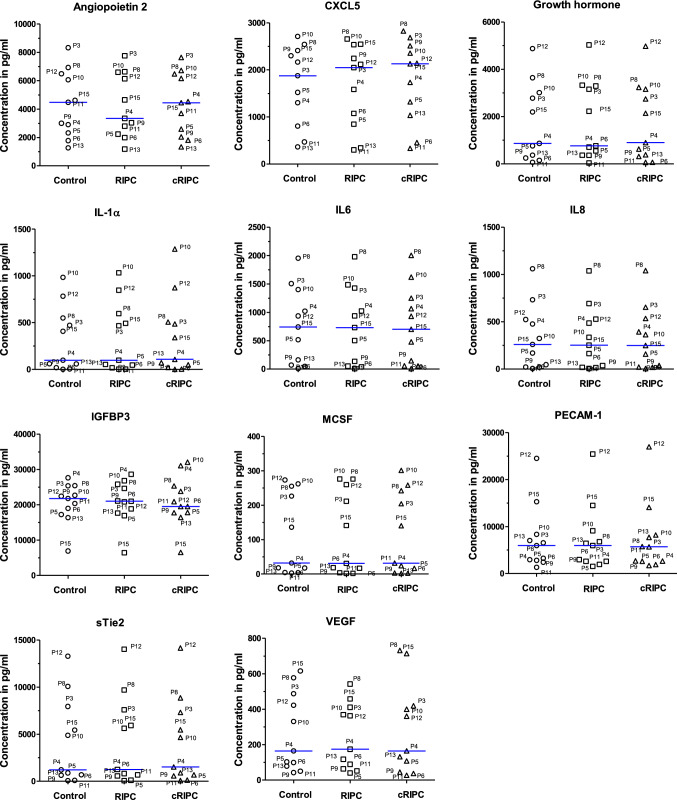
Effects of RIPC/cRIPC on plasma concentrations of 11 selected cytokines. Blue horizontal lines denote the median of the respective group. Values of each volunteer in each group represent the mean of 4 measurements

Based on the proteome profiler results and recent studies of other groups [13, 38], in a second step plasma (Control, RIPC, and cRIPC) from each volunteer was analyzed individually for the concentrations of 11 potentially organ protective cytokines including the top 3 cytokines detected by proteome profiling (CXCL5, Growth hormone, IGFBP3, IL-1α, IL-6, Angiopoietin 2, VEGF, PECAM-1, sTie-2, IL-8, MCSF) using custom made Quantibody® array-based multiplex ELISA systems. All factors investigated showed strong interindividual variations with respect to the measured plasma concentrations already at baseline [e.g. > 10.000-fold for IL-1α (0.06 pg/ml P6 to 984.66 pg/ml P10; Fig. 3). Hence, RIPC/cRIPC treatment did not lead to statistically significant changes of the median plasma concentrations of any of the 11 selected factors when all volunteers were included and analyzed in a holistic approach (comparison of medians in Fig. 3). An in-depth analysis of the individual subjects, however, revealed a significant increase in plasma levels of some of the factors after RIPC/cRIPC treatment [E.g. CXCL5: P3; Control vs. cRIPC and RIPC vs. cRIPC, both *P* < 0.001; P4; Control vs. RIPC and Control vs. cRIPC, both *P* < 0.01; P8; Control vs. cRIPC, *P* < 0.05; P13; Control vs. cRIPC and RIPC vs. cRIPC, both *P* < 0.001; Growth hormone: P6; Control vs. RIPC, *P* < 0.001; IL-1α: P10; Control vs. cRIPC and RIPC vs. cRIPC, both *P* < 0.001; Fig. 3].

These results tempted us to focus on these three factors (CXCL5, Growth hormone and IL-1α) in further analyses. Raw data, detailed analyses and descriptive statistics for all samples are shown in the Supplemental Data File 3.

Regarding the plasma concentrations of the above mentioned 11 factors, an interesting observation worth mentioning is the existence of several strong linear correlations for some of the factors investigated (e.g. IL-1α, MCSF and sTie-2; Supplemental Fig. 4). Whether this is a coincidental finding or of physiological relevance remains elusive in this study.

### Effects of CXCL5, IL-1α and Growth hormone on in-vitro angiogenesis

**Fig. 4:**
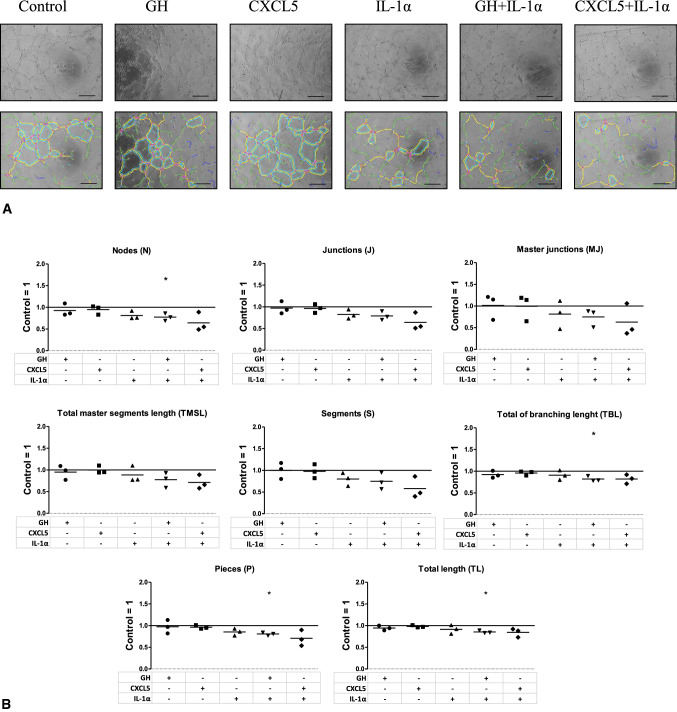
Effects of CXCL5, IL-1α and Growth hormone on in-vitro angiogenesis. **A** representative images of HUVEC cultures that were incubated with Growth hormone, CXCL5, IL-1α, Growth hormone + IL-1α and CXCL5 + IL-1α. Upper panel, original images. Lower panel, results of the graphical analysis using the angiogenesis analyzer tool of the Image J software 1.41 (NIH) [4]. Scale bars depict 300 µm. **B** effects of Growth hormone, CXCL5, IL-1α, Growth hormone + IL-1α and CXCL5 + IL-1α on key parameters of tube formation in-vitro. Scale bars depict 300 µm; horizontal lines show the mean. *, *P* < 0.05; one-sample *T* test. GH, Growth hormone

CXCL5, IL-1α and Growth hormone showed regulation by RIPC/cRIPC in the plasma of some volunteers and CXCL5 as well as Growth hormone also represent factors that revealed regulation by RIPC/cRIPC in proteome profiling assays (Supplemental Fig. 2). In addition, Gedik et al. suggested that IL-1α fulfills the criteria which would be expected from a substance to be released in response to RIPC and to protect the myocardium [13]. Therefore, in a further step, we investigated whether CXCL5, IL-1α and Growth hormone alone or in combination are able to influence in-vitro angiogenesis represented by tube formation potency of endothelial cells (HUVEC). Concentrations of all factors were selected based on the highest value in RIPC/cRIPC plasma measured by multiplex ELISA (CXCL5: 2.8 ng/ml; IL-1α:1.3 ng/ml; Growth hormone: 5.0 ng/ml). In contrast to RIPC/cRIPC plasma which increased several parameters of in-vitro angiogenesis (number of nodes, *P* < 0.05; number of master junctions, *P* < 0.05; number of segments, *P* < 0.05; Fig. 2), this effect could not be mimicked by the addition of CXCL5, IL-1α and Growth hormone alone or in combination. The combination of high concentrations of IL-1α with Growth hormone even had a negative impact on several in-vitro tube formation parameters (Fig. 4).

### Effects of RIPC/cRIPC on surface expression of Tie-2 and CCR2 on circulating monocytes

**Fig. 5:**
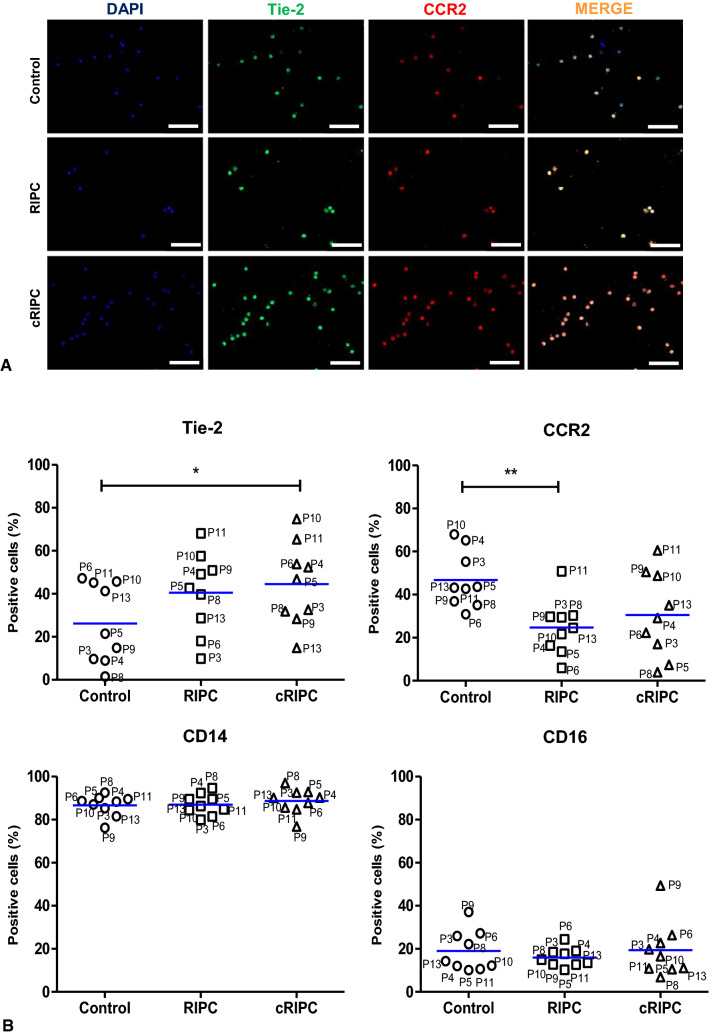
Effects of RIPC/cRIPC on surface expression of Tie-2 and CCR2 on circulating monocytes. **A** representative fluorescent images (DAPI, Tie-2, CCR2 and merge) of human monocytes. **B** percentage of monocytes positive for Tie-2, CCR2, CD14 and CD16 in the Control, RIPC and cRIPC group. Scale bars depict 100 µm; Blue horizontal lines denote the median of the respective group; *, *P* < 0.05; **, *P* < 0.01

CD14 and CD16 are typical cell surface markers of monocytes and define different monocyte populations [51]. Tie-2 (angiopoietin receptor) as well as CCR2 (monocyte chemoattractant protein-1 receptor) expressing monocytes have been described to be involved in inflammation, angiogenesis and tissue repair [8, 11]. Although we did not detect significant effects of supernatants derived from RIPC/cRIPC monocytes on in-vitro angiogenesis, immunofluorescent staining suggested that monocytes of the Control, RIPC and cRIPC group are positive for Tie-2 and CCR2 (Fig. 5A). Detailed analyses using flow cytometry revealed an increase in the number of Tie-2 positive monocytes after RIPC/cRIPC (Control: 26.21% ± 18.49%; RIPC: 40.52% ± 18.78%; cRIPC: 44.55% ± 19.19%. Control vs. cRIPC, *P* < 0.05). The number of CCR2 positive monocytes was decreased by RIPC/cRIPC (Control: 46.77% ± 13.17%; RIPC: 24.71% ± 12.81%; cRIPC: 30.52% ± 19.84%. Control vs. RIPC, *P* < 0.01). Numbers of CD14 and CD16 positive cells were not affected by RIPC/cRIPC (CD14: Control: 86.59% ± 4.98%; RIPC: 86.99% ± 4.91%; cRIPC: 88.67% ± 5.84%. CD16: Control: 19.07% ± 9.48%; RIPC: 15.98% ± 4.32%; cRIPC: 19.36% ± 12.97%).

## Discussion

Our pilot study describes effects of RIPC and cRIPC on humoral factors in plasma as well as on cell surface characteristics of circulating monocytes of a cohort of healthy study participants. In addition, we also investigated the effects of RIPC/cRIPC plasma and plasma proteins (CXCL5, IL-1α and Growth hormone) on in-vitro angiogenesis.

The study design consisted of a daily RIPC treatment for a total of 7 consecutive days and plasma as well as circulating monocytes were obtained at baseline (before RIPC), 3 h after the first RIPC treatment and 3 h after the last RIPC treatment on day 7. Three hours was chosen for the first sampling after RIPC as several authors have shown that the time course of ischemic conditioning, and RIPC in particular, includes two windows of protection. Depending on the study, the first window of protection appears instantly after the conditioning stimulus and lasts between 1 and 3 h, while the second phase of protection appears 12–24 h later [21, 24, 39]. Moreover, results from a study in which plasma-dialysates from healthy volunteers (baseline, 5 min, 30 min, 1 h, 6 h, and daily from 1 to 7 days after RIPC) were infused into Langendorff-perfused mouse hearts subjected to global I/R suggest that RIPC induces the release of cardioprotective mediators within 5 min, and that these factors circulate for up to 6 days [25].

Recent studies imply that daily RIPC (cRIPC) might be more effective for cardiovascular protection than a single RIPC application [6]. Although is tempting to postulate a “dose dependence” of RIPC and conclude that cRIPC can increase the protective effect of a single RIPC treatment, it has to be noted that the safety of multiple ischemic conditioning episodes also termed “hyperconditioning” -examples of “clinical hyperconditioning” are angina pectoris and intermittent claudication- has not been thoroughly examined. In this context it has been proposed that over-dosing of non-lethal I/R, in the form of a large number of I/R episodes, results in a loss of conditioning benefits, and even adverse effects such as collagen damage (i.e. fiber breakage [48]) possibly resulting in negative clinical consequences.

### Effects of RIPC/cRIPC plasma and supernatants derived from RIPC/cRIPC monocytes on in-vitro angiogenesis

In the present work, tube formation assays utilizing human umbilical vein endothelial cells (HUVEC) were performed to estimate the pro-angiogenic capacity of RIPC/cRIPC plasma and cell culture supernatants derived from RIPC/cRIPC treated human monocytes. Although in-vitro tube formation assays do not fully resemble all aspects of in-vivo angiogenesis, they have been used by several groups and represent a reproducible and stable system for the in-vitro analysis of early processes of angiogenesis [3, 22, 23, 42]. Employing in-vitro tube formation assays in combination with computer-assisted analysis [4], several parameters of angiogenesis were significantly increased by RIPC/cRIPC plasma. These results confirm other studies reporting protective effects of RIPC plasma and plasma components on endothelial cells and in-vitro angiogenesis [5, 47]. In contrary to RIPC/cRIPC plasma*,* cell culture supernatants from RIPC/cRIPC monocytes did not significantly influence any of the parameters of angiogenesis investigated. In one of our previous studies cell culture supernatants from human monocytes that were subjected to 3 h of in-vitro hypoxia even negatively affected tube formation in-vitro as a surrogate parameter for angiogenesis [22]. These findings suggest that prolonged hypoxia/ischemia may have detrimental effects on human monocytes and may attenuate potentially positive effects of monocytes on angiogenesis.

### Effects of RIPC/cRIPC on plasma cytokines

Regarding RIPC mediated cardioprotection it is commonly accepted that neuronal as well as humoral signal transfer both play an important role [27]. Gedik et al. have determined plasma concentrations of 25 different cytokines, growth hormones, and other factors before/after RIPC and before/after ischemic cardioplegic arrest in CABG patients. The authors show that only IL-1α may fulfill the criteria which would be expected from a substance to be released in response to RIPC and to protect the myocardium during ischemic cardioplegic arrest [13]. Moreover, Honda et al. demonstrated in a mouse model of septic cardiomyopathy, that RIPC and even more effectively cRIPC for 10 days preserved left ventricular function, improved survival and reduced serum levels of TNF-α, IL-1 and IL-6 [21].

Our proteome profiling results obtained with pooled plasma samples from healthy volunteers suggested that RIPC and cRIPC increases the overall levels of cytokines and in particular the levels of CXCL5, Growth hormone, and IGFBP3, with the tendency of cRIPC being somewhat more effective than a single RIPC application. Based these results and recent studies of other groups [13, 38], 11 candidate molecules were selected (CXCL5, Growth hormone, IL-1α, IL-6, IGFBP3, Angiopoietin 2, VEGF, PECAM-1, sTie-2, IL-8, MCSF) and analyzed by multiplex ELISA systems. RIPC/cRIPC treatment did not lead to significant changes of median plasma concentrations of any of the 11 selected factors when all volunteers were included and analyzed in a holistic approach. This result is not unexpected, considering interindividual variations of up to 10.000-fold even at baseline (Control) with respect to the plasma concentrations of almost all factors investigated. An in-depth analysis of the individual subjects, however, revealed several significant differences especially in the plasma levels of CXCL5, Growth hormone and IL-1α after RIPC/cRIPC treatment in some of the volunteers. CXCL5, a small cytokine belonging to the CXC chemokine family has been described as classical pro-inflammatory cytokine which is involved in tissue remodeling as well as angiogenesis [44]. Some authors also suggested cardioprotective characteristics of CXCL5 which are possibly related to the described effects of CXCL5 on angiogenesis [41]. Similarly, activation of the Growth hormone axis seems to be critically related to cardioprotective effects after myocardial infarction [24]. Regarding IL-1α, Gedik et al. showed an increase in plasma levels after RIPC in patients subjected to elective coronary artery bypass graft (CABG) surgery and suggested IL-1α as a potential factor to be released in response to RIPC and to protect the myocardium [13]. An interesting difference between our results and the data of Gedik and coworkers is that the interleukin levels measured in plasma (especially IL-1α and IL-8) were up to 20-fold higher at baseline and showed greater interindividual variations compared to the values measured by Gedik et al. Methodological reasons seem rather unlikely as both studies employed ELISA/multiplex ELISA, suggesting that differences in the patient/volunteer population used in the two studies could be responsible for the observed differences. While our study included mainly young (mean age 27 years) and healthy subjects, Gedik's work was based on a collective of older (> 60 years) patients with numerous risk factors and comorbidities. Based on these substantial differences, it is even more remarkable that in both studies IL-1α emerged as a possible RIPC-mediated factor.

### Effects of CXCL5, Growth hormone and IL-1α on in-vitro angiogenesis

CXCL5, Growth hormone and IL-1α showed regulation by RIPC/cRIPC in the plasma of some volunteers and CXCL5 as well as Growth hormone also represent factors that revealed regulation by RIPC/cRIPC in proteome profiling assays. As Gedik et al. suggested that IL-1α fulfills the criteria of a humoral factor that is released in response to RIPC and may protect the myocardium [13], we decided to investigate whether CXCL5, Growth hormone and IL-1α alone or in combination are able to influence in-vitro angiogenesis represented by tube formation potency of endothelial cells. Concentrations of all factors were based on the highest value detected by multiplex ELISA in plasma of the RIPC/cRIPC group. In contrast to RIPC/cRIPC plasma which increased several parameters of in-vitro angiogenesis, this effect could not be mimicked by the addition of CXCL5, IL-1α and Growth hormone alone or in combination.

The failure to identify pro-angiogenic effects for one or more plasma factors associated with RIPC/cRIPC once more suggests a high complexity of the associated mechanisms, coupled with an individualized response to the RIPC/cRIPC stimulus. This complexity of RIPC associated mechanisms is also supported by recent studies suggesting that besides leukocytes which are fundamental for the initiation of healing processes (e.g. angiogenesis, extracellular matrix remodeling) and preservation of ventricular function [36, 45, 48] erythrocytes, platelets, and other cell types can release microvesicles and exosomes which may have both detrimental or protective characteristics in the setting of I/R [9]. Interestingly, some authors also described an important role of the vago-splenic axis in RIPC mediated organ protection. The immediate activation of the spleen, which acts as reservoir of inflammatory monocytes/macrophages, through vagal nerves releases cardioprotective factors which reduce infarct size, whereas a more delayed splenic activation first increases myocardial inflammation and then resolves it, providing an orchestrated myocardial healing response [19, 30].

### Effects of RIPC/cRIPC on cell surface expression of Tie-2 and CCR2 on circulating monocytes

Several studies proposed that direct cell–cell interactions of monocyte/macrophage subtypes with endothelial cells are able to induce angiogenesis [7]. Besides M2 macrophages, Tie-2 expressing monocytes (TEMs) were identified to exhibit strong pro-angiogenic characteristics [8]. TEMs physically interact with endothelial cells leading to the formation of vascular networks and induction of angiogenesis [36].

The results of our study reveal a significant increase in the number of Tie-2 positive monocytes in the cRIPC group and a decrease of CCR2 positive monocytes after RIPC. Patel and colleagues demonstrated that patients with critical limb ischemia (CLI) show elevated level of circulating TEMs, while after surgical revascularization levels of TEMs decrease to values of healthy control patients [38]. In-vitro co-culture experiments revealed that TEMs from CLI patients are able to induce angiogenesis, while Tie-2 negative monocytes fail to induce in-vitro tube formation [38]. Our findings that supernatants from RIPC/cRIPC monocytes do not influence in-vitro angiogenesis go along well with the hypothesis that monocytes (i.e. TEMs) exert their pro-angiogenic effects through physical cell to cell interactions.

CCR2 represents the receptor for monocyte chemotactic protein 1 (MCP-1), which is one of the main chemotactic factors attracting pro-inflammatory monocytes towards the side of inflammation and plays an important role in the development of cardiovascular diseases [11]. While classical pro-inflammatory monocytes express high levels of CCR2, non-classical monocytes and M2-macrophages, which are involved in tissue repair and angiogenesis, typically do not possess CCR2 [11]. The same applies to TEMs, which do not express CCR2. Instead responding to MCP-1, TEMs are guided to areas of active neovascularization by angiopoetin-2/Tie-2 interaction [38]. Our finding of an increased expression of Tie-2 after cRIPC and decreased expression of CCR2 on monocytes after RIPC reveals similarities between the Tie-2 positive pro-angiogenic monocytes in CLI patients and the monocytes described in our study. Therefore, it may be hypothesized that cRIPC leads to the induction of Tie-2 positive pro-angiogenic monocytes which induce angiogenesis by physical interactions (with e.g. endothelial cells or progenitor cells) rather than by secretory products. The observation that cRIPC leads to a statistically significant increase in the number of Tie-2 positive monocytes while RIPC does not supports the previously described hypothesis of other authors that cRIPC may -at least in some aspects- be more effective for cardiovascular protection than single RIPC treatment.

### Limitations of the study

There are some limitations of our study that need to be considered. (I) As several authors have shown that the time course of RIPC comprises 2 windows of protection with the first window appearing instantly after the conditioning stimulus and lasting between 1 and 3 h, we cannot exclude the possibility that the time frame of 3 h after RIPC which has been chosen for the analyses in our study might already represents the upper limit of the duration of the first window of protection and that earlier time points of sample collection would have been preferable [17, 20, 31]. (II) Within the context of our in-vitro study, we cannot make any conclusions regarding the role and significance of other blood components such as platelets, erythrocytes, splenic cells or the role of the vago-splenic axis on angiogenesis and RIPC mediated protection. (III) It cannot be ruled out that the in-vitro angiogenesis model used is not capable of sufficiently representing the complexity of the interconnected RIPC events and is therefore only suitable to a limited extent for making predictions about the effects of individual factors and their combinations. (IV) It has to be noted that our results are based on data that were derived from a rather small group size of 11 healthy subjects. Although the data presented might be of restricted immediate value for clinical practice, they highlight the possibility of augmenting cardioprotective effects of RIPC by the use of cRIPC.

### Conclusion

Data from our study suggest that cardiovascular protection might be mediated by RIPC and cRIPC via a regulation of plasma cytokines as well as changes in cell surface properties of monocytes (e.g. Tie-2). Our study also shows the complexity of the RIPC/cRIPC associated processes which seem to be highly dependent on volunteer/patient characteristics and confounding conditions. Although it can be assumed that RIPC/cRIPC will not have beneficial effects in all subjects of a respective cohort, the method is inexpensive, easy to apply and not associated with any serious side effects. Based on this, it seems logical to further advance basic research on RIPC/cRIPC mechanisms in order to help to identify and select the patient population that will profit from RIPC/cRIPC treatment or to increase its effectiveness by combining RIPC/cRIPC with other pharmacological/surgical interventions.

## Supplementary Information

Below is the link to the electronic supplementary material.

Supplementary file1 (PPTX 444 KB)

Supplementary file2 (PPTX 371 KB)

Supplementary file3 (XLSX 862 KB)

Supplementary file4 (PPTX 140 KB)

## Data Availability

Raw data are available from the corresponding author upon reasonable request.
