# Supplementary figures and images for: Effects of remote ischemic preconditioning (RIPC) and chronic remote ischemic preconditioning (cRIPC) on levels of plasma cytokines, cell surface characteristics of monocytes and in-vitro angiogenesis: a pilot study
Source: Basic Res Cardiol. 2021 Oct 14;116(1):60. doi: 10.1007/s00395-021-00901-8 (PMC8516789; doi:10.1007/s00395-021-00901-8)

## Slide 1
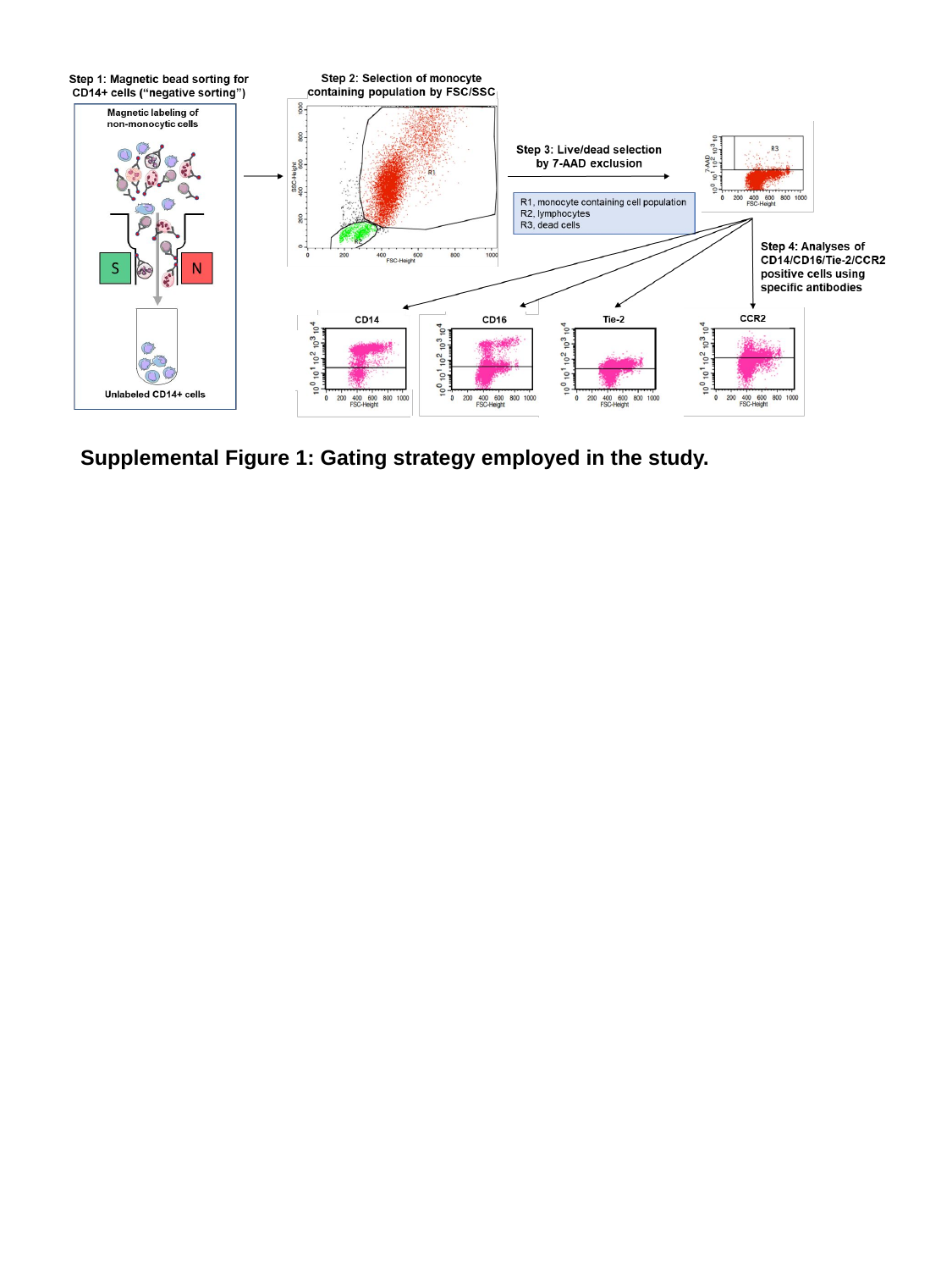

Supplemental Figure 1: Gating strategy employed in the study.

Supplement: Supplementary file 1 — Supplementary file1 (PPTX 444 KB) [file 395_2021_901_MOESM1_ESM.pptx]
