# Supplementary material for: Effects of remote ischemic preconditioning (RIPC) and chronic remote ischemic preconditioning (cRIPC) on levels of plasma cytokines, cell surface characteristics of monocytes and in-vitro angiogenesis: a pilot study
Source: Basic Res Cardiol. 2021 Oct 14;116(1):60. doi: 10.1007/s00395-021-00901-8 (PMC8516789; doi:10.1007/s00395-021-00901-8)
Supplement: Supplementary file 2 — Supplementary file2 (PPTX 371 KB) [file 395_2021_901_MOESM2_ESM.pptx]

## Slide 1
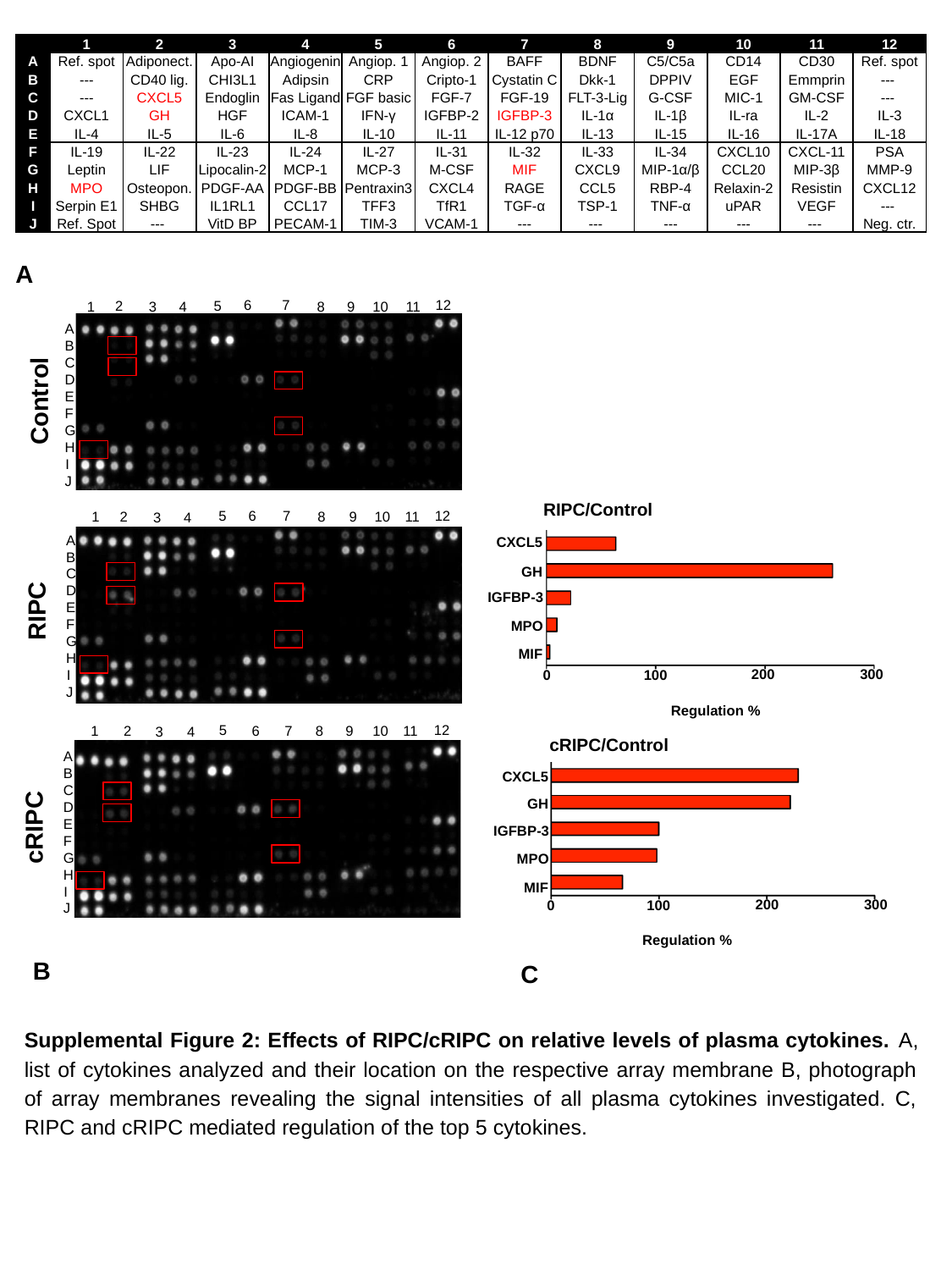

| | 1 | 2 | 3 | 4 | 5 | 6 | 7 | 8 | 9 | 10 | 11 | 12 |
| --- | --- | --- | --- | --- | --- | --- | --- | --- | --- | --- | --- | --- |
| A | Ref. spot | Adiponect. | Apo-AI | Angiogenin | Angiop. 1 | Angiop. 2 | BAFF | BDNF | C5/C5a | CD14 | CD30 | Ref. spot |
| B | --- | CD40 lig. | CHI3L1 | Adipsin | CRP | Cripto-1 | Cystatin C | Dkk-1 | DPPIV | EGF | Emmprin | --- |
| C | --- | CXCL5 | Endoglin | Fas Ligand | FGF basic | FGF-7 | FGF-19 | FLT-3-Lig | G-CSF | MIC-1 | GM-CSF | --- |
| D | CXCL1 | GH | HGF | ICAM-1 | IFN-γ | IGFBP-2 | IGFBP-3 | IL-1α | IL-1β | IL-ra | IL-2 | IL-3 |
| E | IL-4 | IL-5 | IL-6 | IL-8 | IL-10 | IL-11 | IL-12 p70 | IL-13 | IL-15 | IL-16 | IL-17A | IL-18 |
| F | IL-19 | IL-22 | IL-23 | IL-24 | IL-27 | IL-31 | IL-32 | IL-33 | IL-34 | CXCL10 | CXCL-11 | PSA |
| G | Leptin | LIF | Lipocalin-2 | MCP-1 | MCP-3 | M-CSF | MIF | CXCL9 | MIP-1α/β | CCL20 | MIP-3β | MMP-9 |
| H | MPO | Osteopon. | PDGF-AA | PDGF-BB | Pentraxin3 | CXCL4 | RAGE | CCL5 | RBP-4 | Relaxin-2 | Resistin | CXCL12 |
| I | Serpin E1 | SHBG | IL1RL1 | CCL17 | TFF3 | TfR1 | TGF-α | TSP-1 | TNF-α | uPAR | VEGF | --- |
| J | Ref. Spot | --- | VitD BP | PECAM-1 | TIM-3 | VCAM-1 | --- | --- | --- | --- | --- | Neg. ctr. |
A
6
7
12
5
2
8
9
1
10
11
3
4
ABCDEFGHIJ
Control
RIPC/Control
6
7
12
5
2
8
9
1
10
11
3
4
ABCDEFGHIJ
CXCL5
GH
IGFBP-3
RIPC
MPO
MIF
200
300
0
100
Regulation %
12
5
2
6
8
9
1
10
11
7
3
4
cRIPC/Control
ABCDEFGHIJ
CXCL5
GH
cRIPC
IGFBP-3
MPO
MIF
200
300
0
100
Regulation %
B
C
Supplemental Figure 2: Effects of RIPC/cRIPC on relative levels of plasma cytokines. A, list of cytokines analyzed and their location on the respective array membrane B, photograph of array membranes revealing the signal intensities of all plasma cytokines investigated. C, RIPC and cRIPC mediated regulation of the top 5 cytokines.
