# Supplementary material for: Effects of remote ischemic preconditioning (RIPC) and chronic remote ischemic preconditioning (cRIPC) on levels of plasma cytokines, cell surface characteristics of monocytes and in-vitro angiogenesis: a pilot study
Source: Basic Res Cardiol. 2021 Oct 14;116(1):60. doi: 10.1007/s00395-021-00901-8 (PMC8516789; doi:10.1007/s00395-021-00901-8)
Supplement: Supplementary file 4 — Supplementary file4 (PPTX 140 KB) [file 395_2021_901_MOESM4_ESM.pptx]

## Slide 1
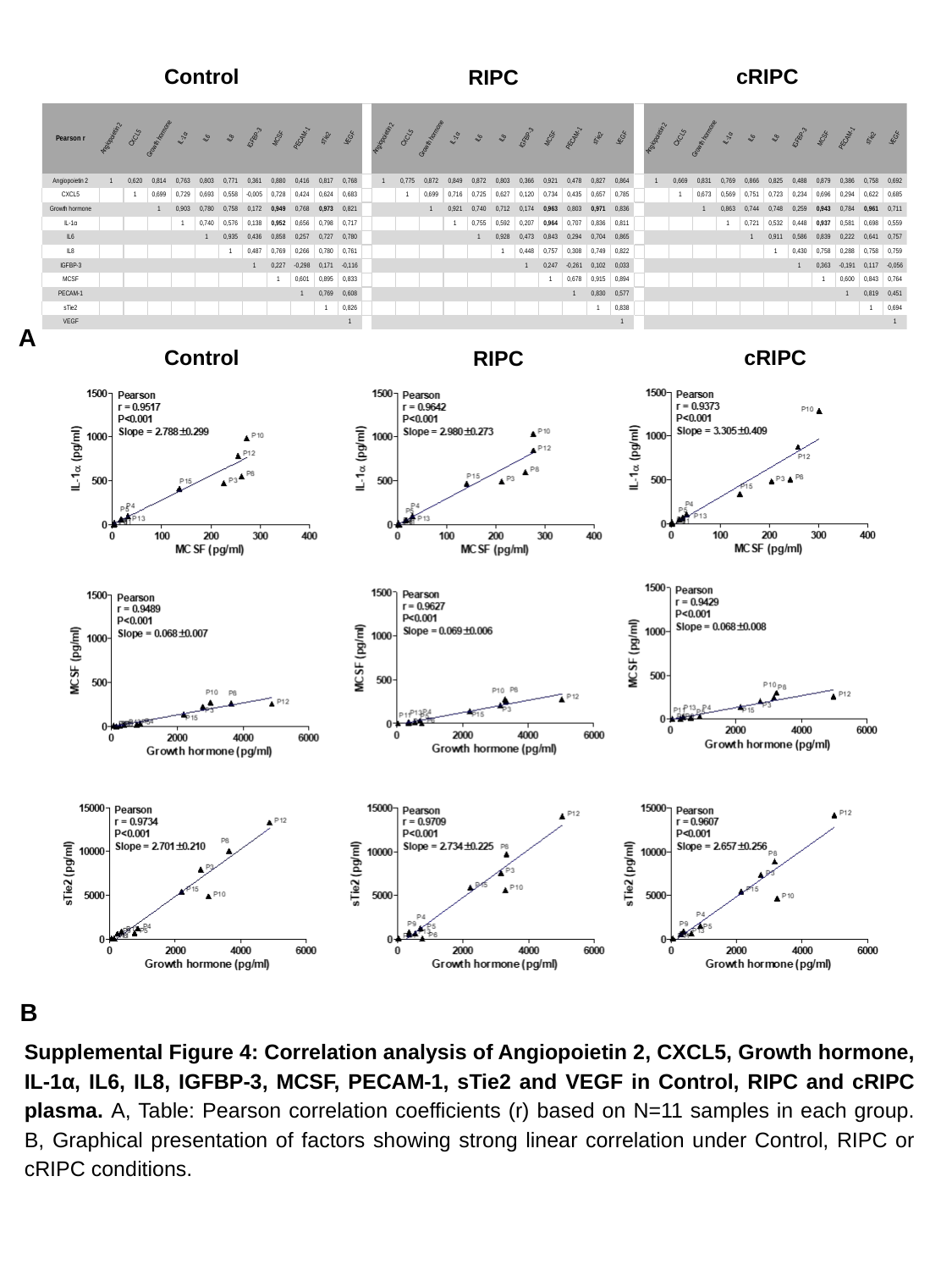

Control
cRIPC
RIPC
A
Control
cRIPC
RIPC
B
Supplemental Figure 4: Correlation analysis of Angiopoietin 2, CXCL5, Growth hormone, IL-1α, IL6, IL8, IGFBP-3, MCSF, PECAM-1, sTie2 and VEGF in Control, RIPC and cRIPC plasma. A, Table: Pearson correlation coefficients (r) based on N=11 samples in each group. B, Graphical presentation of factors showing strong linear correlation under Control, RIPC or cRIPC conditions.
